# The Digital Therapeutic Alliance and Human-Computer Interaction

**DOI:** 10.2196/21895

**Published:** 2020-12-29

**Authors:** Simon D'Alfonso, Reeva Lederman, Sandra Bucci, Katherine Berry

**Affiliations:** 1 School of Computing and Information Systems University of Melbourne Parkville Australia; 2 Division of Psychology and Mental Health School of Health Sciences University of Manchester Manchester United Kingdom

**Keywords:** therapeutic alliance, digital mental health, affective computing, persuasive computing, positive computing, mobile phone, mHealth

## Abstract

The therapeutic alliance (TA), the relationship that develops between a therapist and a client/patient, is a critical factor in the outcome of psychological therapy. As mental health care is increasingly adopting digital technologies and offering therapeutic interventions that may not involve human therapists, the notion of a TA in digital mental health care requires exploration. To date, there has been some incipient work on developing measures to assess the conceptualization of a digital TA for mental health apps. However, the few measures that have been proposed have more or less been derivatives of measures from psychology used to assess the TA in traditional face-to-face therapy. This conceptual paper explores one such instrument that has been proposed in the literature, the Mobile Agnew Relationship Measure, and examines it through a human-computer interaction (HCI) lens. Through this process, we show how theories from HCI can play a role in shaping or generating a more suitable, purpose-built measure of the digital therapeutic alliance (DTA), and we contribute suggestions on how HCI methods and knowledge can be used to foster the DTA in mental health apps.

## Introduction

### Background

The therapeutic alliance (TA), the relationship that develops between a therapist and a client/patient, is a critical factor in the outcome of psychological therapy [1,2]. As mental health care is increasingly adopting digital technologies and offering therapeutic interventions that may not involve human therapists, the notion of the TA in digital mental health care requires exploration. Although work on the TA is largely the province of clinical psychology, questions pertaining to the relationship between a human user and a therapeutic computing system presumably offer a significant opportunity for input from the field of human-computer interaction (HCI).

The term digital therapeutic alliance (DTA) is a broad one that can be applied to a range of types of digital mental health care or interventions, including computer-mediated teletherapy [3,4], web/mobile apps, and therapy agents driven by artificial intelligence [5-8]. This paper focuses on the notion of a DTA in terms of web and particularly mobile apps for mental health, which predominate the work currently carried out under the banner of digital mental health. It is also where work in HCI can be most directly applied, particularly in the case of smartphone mental health apps. Research on smartphone interfaces and the psychological aspects of interaction between a user and their smartphone as a technological object could inform the development of mental health app features that are conducive to DTA formation. Although some of the work covered in this paper could benefit digital health and behavior change technologies more generally, given the motivations behind this paper and the TA as a psychological or mental health concept, this paper focuses solely on digital mental health interventions.

### Objectives

As our starting point in this discussion piece, we consider the recent efforts to develop quantitative measures of the DTA from measures of the traditional TA. Given the incipience of this topic, little work has been conducted on devising or employing measures of the DTA. Early efforts have more or less taken an existing measure and simply modified the items such that *therapist* is replaced with the word *app* or *program* [9,10]. Even the so-called Working Alliance Inventory for Technology-Based Interventions essentially takes this approach [11]. Although perhaps a convenient starting point, such an approach ultimately seems unsatisfactory as it cannot account for certain nuances, particularities, and complexities that could arise in the context of digital interventions. Furthermore, although there will surely be an overlap between traditional and digital therapy, not all components of the traditional TA will necessarily apply to a DTA. There may also be dimensions of alliance in the digital contexts that are not accounted for in traditional models of the TA. To date, perhaps the most considered and detailed attempt to construct a customized measure of the DTA that does not simply mirror traditional measures comes from the study by Berry at al [12], which adapts the Agnew Relationship Measure (ARM) [13] of the traditional TA for use with mental health apps by appropriately modifying and removing items in accordance with consultations with mental health professionals and clients.

In this paper, we use this Mobile Agnew Relationship Measure (mARM) as a specific starting point by discussing its items in terms of themes or topics in HCI. Despite the positive gains made with the mARM in terms of attempting to devise a custom measure of the DTA, this attempt is solely based on applying user feedback and considerations, obtained from a clinical psychology environment, to inform modifications to an existing measure from clinical psychology. Given the significance of the interaction between humans and machines in digital mental health interventions, we show that scrutinizing the mARM items through a lens of HCI theories can provide a valuable complementary approach to considering the DTA, which could inform further work on modifying existing measures or even generating measures from scratch.

## The (Digital) Therapeutic Relationship/Alliance

### Conceptualizations

Work on formulating the notion of a therapeutic relationship between a client and a human therapist emerged over the course of the 20th century with the development of psychotherapeutic practice. For example, Carl Rogers, a pioneer of the humanistic approach to psychotherapy, argued that the primary task of the therapist was to embody 3 core conditions required for therapeutic change to occur: empathy, unconditional positive regard (acceptance) for the client, and congruence (what a therapist says and does matches what they think and feel) [14-16]. Although for the purpose of this paper, the terms *relationship* and *alliance* have been used more or less interchangeably, in one sense, *relationship* encompasses all aspects of the client-therapist relationship, whereas *alliance* refers to a specific aspect of the relationship by which the client and therapist hope to engage with each other to produce positive therapeutic outcomes [1]. Bordin [17] conceptualized this therapeutic or working alliance as consisting of 3 parts: (1) goals (mutual understanding of what the client hopes to achieve with therapy), (2) tasks (what the therapist and client agree needs to be done to achieve the goals), and (3) bond (the bond of trust and confidence between the client and therapist). The conceptualization by Bordin forms the basis of the Working Alliance Inventory (WAI) scale [1,18,19], the most commonly used measure of the TA in face-to-face therapy. Another conceptualization of the TA, consisting of bond, partnership, confidence, openness, and client initiative categories, forms the basis of the commonly used ARM [13].

Despite a history of research showing that the quality of the client-therapist alliance is a significant factor in the successful outcome of therapy [2], an underexamined point in determining the efficacy of digital mental health apps has been if, and to what extent, a user might develop a therapeutic connection with a mental health app. Even if such a DTA does not directly predict treatment outcomes, the formation of a DTA may support the user persisting with the app rather than prematurely discontinuing its use [20]. Research in the digital mental health field has, until recently, largely ignored the concept of a DTA when running clinical trials and developing digital mental health tools; however, given the implications of the impact of the DTA on engagement and outcomes, it is vital that researchers explore this concept in further detail. In fact, the DTA was voted as one of the top 10 research priorities in a 2018 national study in the United Kingdom involving over 600 mental health stakeholders [21].

### Quantitative Measures of the DTA

As has been established, it is only recently that interest in the DTA has seen the emergence of a couple of efforts to devise measures of the DTA that go beyond simply using existing measures of the TA and modifying items such that *therapist* is replaced with the word *app* or *program*. In considering the DTA, Henson et al [22] took the WAI and its 3 categories of goals, tasks, and bond and informally constructed a short 6-item Digital Working Alliance Inventory, breaking each of the 3 categories into 2 app features judged essential for a client-app alliance to be formed (Table 1).

**Table 1:** Items of the Digital Working Alliance Inventory.

Number	Item	Category
1	“I trust this app to guide me toward my personal goals”	Goals
2	“I believe these app tasks will help me to address my problem”	Tasks
3	“This app encourages me to accomplish tasks and make progress”	Bond
4	“I agree that the tasks within this app are important for my goals”	Goals
5	“This app is easy to use and operate”	Tasks
6	“This app supports me to overcome challenges”	Bond

To date, however, the mARM [12], an adaptation of the ARM, is to the best of our knowledge the most considered and detailed attempt to derive a custom measure specifically for digital mental health apps. The development of the measure involved 3 stages:

1. Interviews with mental health clients about the concept of TA in the context of a digital health intervention to derive key themes from interview transcripts using thematic analysis.
2. Rating scales and open-ended questions to elicit views from clients and mental health staff about the content and face validity of the original ARM scale that replaced the word therapist with the word app.
3. Findings from stages 1 and 2 used to develop the mARM, employing a decision-making algorithm about the items to be dropped, retained or adapted.

A list of the items in the mARM is provided in Table 2.

It is worth noting that the following original ARM items were deemed irrelevant and removed from the mARM:

- “I am worried about embarrassing myself when using the app”
- “The app feels persuasive”
- “The app seems bored”
- “The app and I have difficulty working jointly as a partnership”

These omissions seem to make sense. Clients might be worried about embarrassing themselves with a human therapist, yet it does not seem possible to be concerned about embarrassing oneself in the eyes of an app per se. Similarly, smartphones cannot become bored and one does not enter into partnerships as such with an app. The item concerning persuasion, however, is an interesting one, since, as we will discuss below, there is a whole field that concerns itself with persuasive technology design.

**Table 2:** Items of the Mobile Agnew Relationship Measure.

Number	Item	Category
1	“I feel free to express the things that worry me”	Openness
2	“I feel friendly towards the app”	Bond
3	“I take the lead when using the app”	Client initiative
4	“I hold back some important things about myself from the app”	Openness
5	“I have confidence in the app and the things it suggests”	Confidence
6	“I feel optimistic about my progress”	Confidence
7	“I feel I can openly express my thoughts and feelings when using the app”	Openness
8	“I feel disappointed in the app”	Confidence
9	“I can share personal matters I am normally ashamed or afraid to reveal”	Openness
10	“I look to the app for solutions to my problem”	Client initiative
11	“I have confidence in the app and how it works”	Confidence
12	“The app accepts me no matter how I respond”	Bond
13	“The suggestions the app makes are important to me”	Confidence
14	“The app seems to understand me”	Bond
15	“The app feels warm and friendly with me”	Bond
16	“The app does not give me the help I would like”	Confidence
17	“The app is supportive”	Bond
18	“The app seems to ignore my needs”	Partnership
19	“The app confidently presents its information”	Confidence
20	“I am responsible for my recovery, not the app”	Client initiative
21	“The more I use the app, the more I get out of it”	Partnership
22	“The app gives me the confidence to take the lead in my recovery”	Client initiative
23	“I agree with the direction the app is taking me”	Partnership
24	“The app is like having a member of my care team in my pocket”	A novel addition (not resulting from retention or adaption) to capture a key theme from interviews
25	“I am clear about what the app can and cannot offer me”	Not categorized in the Agnew Relationship Measure

## Applications of HCI

The field of HCI concerns the design of computer technology and how humans interact with such technology, particularly how it can be best designed to facilitate its use [23]. When considering digital mental health apps and the role HCI might play in DTA formation, it is not necessarily about ways in which the smartphone or computer can be anthropomorphized. Rather, we are interested in the smartphone or computer as a device per se and the ways in which apps can be given features, including those that make use of certain capacities, particularly in the case of smartphones, to foster the DTA. For example, a theme that will be considered as this paper unfolds is how the power afforded by modern smartphones to infer user behavior and context [24,25] might offer new opportunities to personalize content and tailor responses to support fostering of a DTA. We will now take a look at several areas of HCI germane to the DTA, before an extended discussion of how these areas can apply to questions relevant to the DTA via mARM items.

### Persuasive System Design

A persuasive computing technology is “a computing system, device, or application intentionally designed to change a person’s attitudes or behaviour in a predetermined way” [26]. Fogg coined the term *captology* from the phrase Computers as Persuasive Technologies [26-28] to reflect this idea. As we will now briefly elucidate, persuasive design principles are relevant to several DTA criteria, not just the direct matter of whether *the app feels persuasive*.

Informed by Fogg’s conceptualization of persuasive technology Oinas-Kukkonen and Harjumaa [29] have developed a concrete framework that transforms persuasive design principles into software requirements and system features. According to their persuasive systems design (PSD) model, there are 4 categories for persuasive system design, each consisting of several principles:

1. Primary task support: the design principles in this category support the execution of the user’s primary task and consist of reduction, tunneling, tailoring, personalization, self-monitoring, simulation, and rehearsal.
2. Dialogue support: the design principles in this category are about the feedback an interactive system provides to its users to help them move toward their goal or a target behavior. This category consists of praise, rewards, reminders, suggestion, similarity, liking, and social roles.
3. System credibility support: the design principles in this category describe how to design a system so that it is more credible and thus more persuasive. The category consists of trustworthiness, expertise, surface credibility, real-world feel, authority, third-party endorsements, and verifiability.
4. Social support: the design principles in this category describe how to design the system so that it motivates users by leveraging social influence. The category consists of social facilitation, social comparison, normative influence, social learning, cooperation, competition, and recognition.

Of these 4 categories, the fourth one is not fully relevant, as this investigation focuses on the connection between an individual user and an app. If a second human were involved, it would generally be a therapist accompanying the app user. However, there are cases of digital mental health interventions involving dedicated social components [30], particularly social networking, in which case social support becomes a significant factor. Several of the mARM items, such as “I feel free to express the things that worry me” and “The more I use the app, the more I get out of it” would vary in meaning given a social (networking) component. We will also be touching upon social relatedness as a psychological principle further on.

Of the first 3 pertinent categories, a selection of principles is particularly relevant when considering the DTA. From Primary task support, the principles of personalization and tailoring require that systems provide personalized content and services and tailored information to users and user groups. Not surprisingly, “personalising tasks or goals to the individual is likely to support the formation of a relationship with the technology” [16], and in subsequent sections, we will discuss how approaches in HCI can be used to foster these aspects of the DTA and items such as “I have confidence in the app and the things it suggests” and “the suggestions the app makes are important to me.”

Several of the dialogue support principles are particularly relevant. Praise (offering praise), rewards (rewarding target behaviors), and reminders (reminding users of their target behavior) are principles whose implementation would support the mARM item *the app is supportive*. The principle of similarity, which says that “people are more readily persuaded through systems that remind them of themselves in some meaningful way” [29] is not just conducive to system persuasiveness but can also make a contribution to the DTA by supporting items such as the app seems to understand me. Finally, several of the system credibility support principles are also particularly relevant. Trustworthiness, expertise (system should provide information showing knowledge, experience, and competence), and surface credibility (system should have competent look and feel) clearly connect with DTA criteria such as “I have confidence in the app and how it works” and “I have confidence in the app and the things it suggests.”

In light of this discussion on persuasive system design, the choice to remove the item *the app feels persuasive* from the mARM because of “low relevancy” and “no alternative options were suggested or agreed upon” [12] is seriously brought into question. Indeed, the issue with this item is a prime example of how investigating the DTA from an HCI perspective can help to shape its conceptualization and measurement.

### Affective Computing

Affective computing is a subfield of HCI that concerns systems and devices that can recognize, interpret, process, and simulate human affects/emotions [31,32]. Advances in smartphones have paved the way for rich opportunities in using data acquired from embedded smartphone sensors and smartphone use to infer a user's affective states [33]. Regarding the DTA, the capacities of affective computing can work in 2 ways.

The first method is to detect a user’s state and tailoring components of the app, such as therapy recommendations and screen messages or information accordingly. States or difficulties such as low mood, anxiety, and stress can be inferred with a variety of technological modalities, including phone interactions, movement sensors, facial analysis, voice analysis, and text analysis [34,35]. Whether it is in immediate response to a momentary signal given off by an individual or from behavior inferred over a longer time period such as a day or a week, an app can use such information to deliver a momentary interventional exercise suggestion, strategy, or message of encouragement [36,37]. For example, the detection of relatively high levels of negative emotional states such as anxiety or stress on a given day, using computational linguistic and acoustic analysis of an individual’s textual and vocal smartphone communications [38,39], could trigger an evening push notification with stress or anxiety management exercises.

The second way concerns simulating affect, in particular via screen content and messages, in such a way that they are appropriate or attempt to induce the right affective state in a user. In general terms, beyond quality content, the form in which the content is delivered is also a factor. Particularly in the cases of virtual agents and bots, there is a reasonable assumption that users would prefer an agent that exhibits in greater quantities the emotional intelligence and general anthropomorphic characteristics of a human therapist. This generalization is challenged, however, by the *uncanny valley* phenomenon [40], whereby people develop a sense of unease and discomfort at robots that fall within a certain range of human likeness, neither not too human-like or human-like enough. Furthermore, some research suggests that it is not unequivocal whether users prefer an emotionally demonstrative system over one that is affectively neutral and that this might depend on the personality type of the user [41].

Despite smartphones and personal computers not having an anthropomorphic form, apps on such devices can still incorporate affective qualities. For example, the type of language that an app uses, and for those apps that include voice, the paralinguistic properties of that voice would influence responses to the mARM such as “I feel friendly towards the app” and “The app feels warm and friendly with me.”

### Eudemonic Psychology and Positive Computing

The notion of human psychological well-being is accompanied by a variety of definitions and approaches to measurement. In the tradition of ethical hedonism [42], hedonic approaches to psychological well-being define it broadly as the experience of positive affect. Termed *subjective well-being*, measures based on this approach generally consist “of three components: life satisfaction, the presence of positive mood, and the absence of negative mood, together often summarised as happiness” [43]. Positive emotions and pleasure seeking are undoubtedly important elements of the human condition, but beyond this sense of well-being lies one with ties to the Aristotelian notion of eudemonia, a notion of well-being that goes “beyond the experience of positive emotion into the realms of engagement, meaning, relationships, and human potential” [44].

This broader sense of well-being, termed eudemonic or psychological well-being, encompasses aspects of positive human functioning and flourishing, such as purposeful engagement in life, realization of personal talents and capacities, and enlightened self-knowledge, [45] aspects neglected by accounts that narrowly focus on satisfaction, feeling good, and contentment.

There are several prominent accounts and frameworks based on this conception of well-being. The Self-Determination Theory (SDT) by Ryan and Deci [43,46] posits 3 basic elements that typically foster subjective as well as eudemonic well-being:

1. Autonomy: feeling agency and acting in accordance with one’s goals and values
2. Competence: feeling able and effective
3. Relatedness: feeling connected to others and a sense of belonging.

Similarly, the framework for eudemonic well-being by Ryff and Singer [47] is concerned with 6 core components: self-acceptance, autonomy, personal growth, positive relationships, environmental mastery, and purpose in life.

The positive psychology movement perhaps most conspicuously embodies the ethos of eudemonic or psychological well-being and the promotion of positive function and flourishing [48]. At the base of positive psychology is the PERMA model, which stands for positive emotions, engagement, relationships, meaning, and achievement. Positive psychology also identifies the importance of using “signature strengths every day to produce authentic happiness and abundant gratification” [49], strengths such as connectedness, gratitude, kindness, open-mindedness, perseverance, honesty, and courage [50].

The incorporation of this conception of well-being into the design and development of computing and information systems is embodied in the emerging field of *positive computing*, which addresses how technology can “support wellbeing that encompasses more than just immediate hedonic experience, but also its longer-term eudaimonia, or true flourishing” [51]. This is achieved through the integration of well-being theories and techniques from frameworks such as SDT and positive psychology into such technologies.

For example, the autonomy component of SDT can be supported by offering options and choices over use and not in turn demanding actions from users without their assent [51]. The component of competence can be enhanced by including optimal challenges that are neither too difficult nor too easy, positive feedback, and opportunities for learning [51]. Finally, an aim to foster relatedness can determine approaches taken in the development of digital systems for social connection. For example, direct communication such as wall posts, comments, and web chat is associated with greater relatedness over mere passive consumption of friends’ content [51]. Research [52] suggests that users develop a quality relationship or bond with health apps that are sensitive to their needs for autonomy and relatedness. Furthermore, listed below are the 5 identified dimensions of autonomy [53] “that are useful for understanding the mediating role that health and wellbeing apps have on the communication of information” [54]:

1. Degree of control and involvement that the user has within the app
2. Degree of personalization over the app’s functionality
3. Degree of truthfulness and reliability related to the information presented to the user and how this affects their decisions
4. User’s self-understanding of the goal pursuit and whether the app promotes or hinders a user’s awareness of their own agency
5. Whether the app promotes some form of moral deliberation or moral values in the actions it recommends.

The implementation of features conducive to the strengths of positive psychology is another example of positive computing. For example, designers might add a *thanks* button based on the evidence that expressing gratitude promotes overall well-being [55]. Furthermore, apps and software built from scratch to promote well-being, particularly digital mental health interventions, can be exemplars of positive computing. For example, the moderated online social therapy (MOST) mental health platform has been built on a basis significantly influenced by positive psychology [56]. Previous work on MOST suggests that platform design informed by the principles of SDT supports the emergence of a DTA between users of a digital mental health platform and the platform itself [57].

In our subsequent discussion, we will point out where approaches to well-being, such as SDT and positive psychology, and hence their HCI embodiment in positive computing, can promote certain DTA items as given in the mARM.

### The Human-Smartphone Connection

The relationships we have with our technological devices such as the smartphone, whether positive or negative, is another relevant field of inquiry, which will primarily relate to the *bond* category of the TA. Smartphone attachment theories may play a role in our understanding of the extent to which humans can develop relationships, such as a DTA, with digital mental health apps. In developing smartphone mental health apps, “it is important to consider that the quality of an individual’s relationship to his/her mobile phone may influence their receptivity to, and ultimately the efficacy of, mobile health (mHealth) programs and interventions” [58].

Problematic mobile phone use and smartphone addiction are phenomena that are gaining some diagnostic currency [58,59]. There is even a purported phenomenon that goes by the neologism nomophobia, a portmanteau derived from NO MObile PHone PhoBIA, which also has a questionnaire to quantitatively measure it [60]. Smartphone addiction scale items such as “having my smartphone in my mind even when I am not using it” [59] are indicators of a negative relationship. However, they suggest the possibility of a deep bond between the user and phone, which, if combined with positive goal development, could be harnessed for beneficial, therapeutic ends.

The study by Ribak [61] has described how mobile phones can act as transitional objects for adolescents. Furthermore, Vincent [62] explores and examines the concept of emotional attachment to mobile phones, and Melumad and Pham [63] show that smartphones can serve as attachment objects for consumers:

Results from two experiments show that smartphones provide greater comfort and faster recovery from stress (vs. PCs), defining characteristics of attachment objects. A third study shows that smartphone use becomes pronounced among consumers particularly susceptible to stress – those who recently quit smoking.

As discussed in the study by Li et al [52], there is a tendency for emotional bonding and attachment behaviors toward a health app to occur when the app user is experiencing something negative and the app attends to their basic needs, such as providing help with ill-health. Such emotional bonding is conceptualized as an affectionate response when individuals use health apps, which manifests in 3 aspects: “warm feelings when using mHealth apps; they become aroused with intense and positive moods about mHealth apps; and they sense close connections with mHealth apps” [52]. This connection between a user and their smartphone would pertain to bond mARM items such as “I feel friendly towards the app.” Furthermore, it would also seem to pertain to the novel item “The app is like having a member of my care team in my pocket.”

Before moving on to the next section, one final point to gather from the discussion of the 4 HCI areas in this section is that the psychology or personality of the individual user is likely to play a role in which apps or app features work for them in terms of DTA formation and app adoption more generally. This indicates an advantage for recruitment systems whereby a preunderstanding of the user can be used to establish the suitability of an app for them. Beyond this possibility, which will most often not necessarily be the case, data-based profiling techniques embedded into app technologies provide another way to learn about the user on the fly and establish their relevant personality characteristics.

## Assessing mARM Items in Light of HCI

We now examine each of the 25 mARM items listed below and discuss, where applicable, what the HCI topics of PSD, affective computing, positive computing, and the human-smartphone connection have to say about them, thus facilitating an exploratory discussion of the DTA and HCI via the structure of the mARM. A rough classification of 3 item types emerged, consisting of the following:

1. Items which can be supported by HCI theories.
2. Items for which HCI considerations are not directly relevant or which are not linked to specific HCI topics, as they are more so questions gauging the characteristics of the app user.
3. Items whose inclusion or exclusion in a DTA measure is brought into question in light of HCI considerations.

### 1. I Feel Free to Express the Things That Worry Me

An app to which this item applied would by definition be one in which users can express their worries. For example, the app might contain a simple journaling feature or questions in an exercise designed to elicit responses from the user expressing their worries. Another more involved possibility is the interaction with a conversational feature or agent in the app. In such cases, the suitability of the journaling medium or questions asked would influence the quality of what the user expresses. Trust and what the app does with what the user shares could also influence their expression, including data privacy and security, and whether the responses will be seen by another human. Whatever the case, however, it does seem that a core part of this question does not apply in the case of an app, namely, the freedom or inhibition a client may feel in expressing their worries depending upon the relationship they have with their human therapist. A related, perhaps more apt question could be “I find it beneficial expressing the things that worry me.”

Although the PSD category of social support is not a focus of individual user apps, this item would come to have another significance were the app to have a function through which worries could be expressed with peers and/or clinicians.

### 2. I Feel Friendly Towards the App

This item involves and is an opportunity to emphasize an important point that pertains to several of the mARM items and the DTA in general. Despite interacting with a nonhuman, nonconscious agent, people demonstrate a willingness to form human-like relationships with technology. As early as the 1960s, the tendency to anthropomorphize computers, ascribing to them human traits and intentions that they do not actually have, was observed with ELIZA, an early natural language processing computer program that simulated a Rogerian psychotherapist (dubbed the *ELIZA effect*) [64]. Furthermore, research [65] indicates that “the sophisticated interactions people have with computers engage many of the same cognitive schema and patterns of behaviour found in human social interactions” [16].

The fact that “people reciprocate positive behaviours from computers by behaving similarly in return” [16] suggests that the incorporation of certain affective computing characteristics or qualities in an app would engender a system that supports this item by providing friendly cues and language. However, what in fact this item would be measuring is brought into question by research suggesting that such interactions are *mindless*, that is, people are simply mindlessly following triggered social scripts and responding to computers as social actors via a relatively automatic process beyond their awareness, rather than a conscious choice [16,66].

### 3. I Take the Lead When Using the App

Although this item is largely dependent on the nature of the user, the facilitation of autonomy with supportive, positive computing design and features would be conducive to this item. Facilitating *concordance*, where an individual can modify an intervention to suit the way they prefer to use it, rather than just *adherence*, where the system might prescribe a strict therapy pathway the user should stick to, would also give users more opportunity to take the lead [57].Of the 5 dimensions of app autonomy listed earlier in the section on *Eudemonic Psychology and Positive Computing*, dimensions 1 (degree of control and involvement that the user has within the app) and 2 (degree of personalization over the app’s functionality) would also contribute to this item.

### 4. I Hold Back Some Important Things About Myself From the App

Responses to this item would largely be determined by an individual’s psychology and attitude toward app therapy. However, the extent to which an app demonstrates PSD principles such as trustworthiness and expertise will perhaps influence how many important things about themselves the user is willing to share.

Inducing users to share things with PSD incentives such as personalization (eg, the more you share with the app, the better tailored the app will be for you) and self-monitoring (eg, sharing with the app will provide you with monitoring snapshots about yourself) are other options to promote this item. Further options include employing praise or rewarding users when they share things. However, these latter possibilities, in particular, raise consideration of the spectrum between intrinsic motivations and extrinsic motivations; there is a qualitative difference between a user sharing important things about themselves because it has intrinsic therapeutic value for them versus sharing things because they receive some extrinsic reward in doing so. However, gaining useful information from and about the user so that an app may better serve them, even if the information is obtained via extrinsic reward incentives, is generally better than nothing.

### 5. I Have Confidence in the App and the Things It Suggests

Trustworthiness, expertise, and surface credibility (system should have competent look and feel), design principles in the PSD category of system credibility, would naturally support this DTA item. More interesting are apps that aim to deliver accurate and relevant personalized therapy suggestions for the user. The PSD principle of personalization can be defined as “the ability to provide contents and services tailored to individuals based on knowledge about their needs, expectations, preferences, constraints, and behaviours” [67]. The ubiquity of digital technologies that are equipped with sensors for inferring user behaviors, situations, and contexts, combined with advances in data processing and science, has augmented the possibilities of personalized recommendations and personalized HCI more generally [37,68].

If an app does deliver personalized therapy suggestions based on a user’s app use history or their smartphone sensor information, then an explanation of why the suggestion was made would presumably help to promote a sense of confidence [69]:

Explainable Recommendation refers to the personalized recommendation algorithms that address the problem of why - they not only provide users with the recommendations, but also provide explanations to make the user or system designer aware of why such items are recommended. In this way, it helps to improve the effectiveness, efficiency, persuasiveness, and user satisfaction of recommendation systems.

Recommendation systems and explainability are fascinating and complex topics. Some basic example forms of explained recommendation will suffice to convey this idea, which will already be familiar to those who use websites that deliver content such as Netflix and Amazon:

- Therapy item X was suggested because you have recently completed therapy item Y (with X and Y having a predefined relevance connection).
- Therapy item X was suggested because you told us fact Y about yourself.
- Therapy item X was suggested because users similar to you have benefited from it.

Furthermore, the application of smartphone sensing for contextual awareness and personal sensing insights [35] raises a range of rich recommendation possibilities and explainability challenges. For example, suppose that an individual who generally goes to bed before midnight during weeknights is up at 3 AM using social media on their smartphone for a third consecutive weeknight. This fact, coupled with other recent smartphone use patterns such as keystroke dynamics, might be indicative of stress-related insomnia and could be an opportunity for their mental health app to offer push notifications for a real-time therapy exercise to help with their condition. If so, an appropriately worded explanation for such a recommendation would also need to be considered.

These points serve to make the case that an app, powered by smartphone technology and algorithmic intelligence, would inspire confidence by successfully generating accurate personalized suggestions that resonate with the user and, furthermore, by accompanying them with a good explanation (especially for more sophisticated suggestions).

### 6. I Feel Optimistic About My Progress

In terms of PSD, the dialogue support principles of praise (offering praise) and rewards (rewarding target behaviors) would foster this item.

In addition, unless the mental health app involves social networking or human moderation, the relatedness component of SDT has no direct import. However, certain indirect features could be incorporated into an app to encourage use and engagement with therapy. One can imagine a feature that enables certain successes or milestones within the app to be shared with an individual’s social network channels. Such a feature would support this item.

### 7. I Feel I Can Openly Express My Thoughts and Feelings When Using the App

Most of the points made above for item 1 apply to this item. Whatever means an app has for users to express their thoughts and feelings, it should be easy to do so, and the user must be confident that the content they share will be used appropriately. Similar to item 4, the informational value an app offers the user in response to sharing their thoughts and feelings can also be a fundamental incentive. For example, in the theme of self-tracking with technology and the PSD principle of self-monitoring, “app-based features that enable users to self-monitor their mood by periodically reporting their thoughts, behaviours, and actions can increase emotional self-awareness (ESA)” [70]. This ability to identify and understand one’s own emotions “has been shown to reduce symptoms of mental illness and improve coping skills” [70].

### 8. I Feel Disappointed in the App

A variety of matters, including HCI ones and those involving the theories introduced earlier, can influence responses to this item: usability of the app, quality of the content, nature of the individual using the app, and accuracy and reliability of the app. Another important aspect of this item can be considered a form of congruence for computing systems: how the app presents itself and the experience it delivers should be consistent and match the expectations and relationship the user forms with the app. For example, suppose that an app has an onboarding process asking the user for personal information, with messages that this information will be used to provide the user with relevant information and tailored help throughout their journey with the app. This will set up an expectation in the user that the app will do what it signals it will do, such that if the app fails to deliver on its promise and does not satisfy the user with relevant information or provides egregiously irrelevant personalized suggestions, it is likely to disappoint the user.

### 9. I Can Share Personal Matters I Am Normally Ashamed or Afraid to Reveal

This item obviously shares similarities and overlaps with items 1 and 7. In the case of traditional therapy, the score received for this item will be a function of the relationship developed between the client and therapist. In the case of an app, certain app features might foster this item; however, the score will largely be a function of the user’s attitude toward using mental health apps. Another factor positively contributing to this item is the fact that apps offer users, particularly those concerned about such a thing, a means to share personal matters without (directly) communicating with another person and feeling stigma or concern that what they share will be scrutinized. This aspect of computers has often been mentioned as one of their advantages as a mental health care solution [71,72].

### 10. I Look to the App for Solutions to My Problems

The score an app receives for this item will be a function of both the quality of the app and the user’s willingness to use it as a solution in their mental health care.

### 11. I Have Confidence in the App and How It Works

Trustworthiness, expertise, and surface credibility (system should have competent look and feel), design principles in the PSD category of system credibility, naturally support this DTA item. It should be noted that this mARM item seems related to item 5, and it is worth considering how the distinction between the 2 analogous original ARM items might be affected when translated into mARM form (the item 5 ARM equivalent is “I have confidence in the therapist and their techniques” and the item 11 ARM equivalent is “The therapist’s skills are impressive”). The ARM versions seem sufficiently independent, whereas with the mARM versions, the response to item 5 seems quite constitutive of the response to item 11; an app that does not offer good suggestions is in one important or even crucial sense not working well, despite the fact that the app may be technically impressive. If these 2 items are to remain distinctive in a measure of the DTA, then the item replacing the ARM notion of item 11 could perhaps be something like *the app is technically impressive*.

### 12. The App Accepts Me No Matter How I Respond

This item ties in with the Rogerian notion of unconditional positive regard. The first question for this item concerns the notion of acceptance; it is questionable if a computing device can *accept* the responses of a user in the same way as intended in the original ARM question, though they can provide responses indicating some form of programmed acceptance.

However, an app should not provide blanket responses of acceptance to any user response. Although an app should not reject a user or provide responses containing unnecessary negativity, “negative or directive feedback provides guidance, leading people to become, over time, more certain about their behaviour and more confident in their competence” [73].

### 13. The Suggestions the App Makes Are Important to Me

This item shares some overlap with item 5, and it stands to reason that the PSD principle of personalization of app content delivery and the quality of that content will increase the chances of better scores for this item. Findings of the study by Duggan et al [74] suggest that personalizing tasks or goals to the individual is likely to support the formation of a relationship.

### 14. The App Seems to Understand Me

As with item 13, personalization of app content delivery will increase the chances of better scores for this item. In fact, one qualitative analysis identified that automated personalization helped one user feel understood and that intelligent responses from an app fostered the perception of a relationship for another user [75]. There is also a connection between personalization and user autonomy (first principle of SDT), as “personalization also creates a sense of ownership and choice beneficial to autonomy” [51]. However, there are 2 types of personalization that require distinction. The first type provides users with the ability to customize their experience of the system by giving them access to edit certain settings, in line with app autonomy dimension 2 introduced earlier. The second type is that of automated personalized content recommendation systems. The former of these 2 types is relatively straightforward; however, the latter raises consideration of possible tensions between user autonomy and system automation.

Although most flagrantly problematic in the case of big commercial platforms such as YouTube and Facebook, whose recommendation systems, newsfeed, and advertising are fraught with consequences of political, social, and epistemological detriment, the design of recommendation systems for automated intervention suggestions in health apps, particularly mental health apps, warrants consideration. Beyond the issues of ensuring safe, accurate recommendations possibly accompanied by explanation, recommendation systems can negatively encroach on a users’ autonomy by nudging them in a particular direction or limiting the range of options which could be presented to them, even possibly in extreme cases *addicting* them to certain content or actions [76]. If autonomy and self-directedness are conducive to TA formation and positive therapeutic change, we need to create systems that strike a balance between providing personalized automation and facilitating the client decision making necessary for effective therapy. The app autonomy dimension 5 introduced in the section *Eudemonic Psychology and Positive Computing* (whether the app promotes some form of moral deliberation or moral values in the actions it recommends) is pertinent in these considerations.

### 15. The App Feels Warm and Friendly With Me

How can an app possibly be made to feel warm and friendly? One possibility is for the app to use language that is warm and friendly. However, perhaps more significant would be for the app to have an affective computing ability to detect a user’s affective state and tailor its responses accordingly. As has been noted by others, the Rogerian notion of congruence “is a particular challenge for technological interventions where it is trivial to programme expressions of empathy or positive regard, but not easy to imbue these expressions with genuineness or authenticity” [16].

A smartphone does not have intentionality, and any signaling of empathy is not an embodiment of some consciousness correlate. However, such signals can be generated in such a way that users, in the spirit of the ELIZA effect, treat them as though they do have a degree of genuineness or authenticity. This is by programming the device so that it clearly acts in a way that is sensitive to its environment and its user. An app that exhibits artificial emotional intelligence by responding in such a way will possibly give users a sense of empathy via a simulative effect.

### 16. The App Does Not Give Me the Help I Would Like

General failures of HCI principles, poor information quality, and unsuitable therapy content can all contribute to higher scores for this item. Responses to this item would also largely be a function of the user’s needs and the psychological content of the app.

### 17. The App Is Supportive

Praise (offering praise), rewards (rewarding target behaviors), and reminders (reminding users of their target behavior) are PSD dialogue support principles whose implementation would support this item.

### 18. The App Seems to Ignore My Needs

An app being affectively and effectively responsive will also decrease the chances of dissatisfaction with the app and correspondingly minimize values for this item. When users provide input indicating the need for help, an app needs to readily respond with information or therapy content relevant to the needs of the user.

### 19. The App Confidently Presents Its Information

This item overlaps with item 5. Apps can easily present information, but how can they present information in a manner that a user perceives as confident? Generally speaking, an app that promotes this item should incorporate PSD system credibility support principles such as expertise, surface credibility, authority and verifiability. Signaling to users, where appropriate, that app content being delivered is evidence based is a relatively easy way to help achieve this. In terms of personalized therapy suggestions that an app may present, explanation would signal that the app is confident in the information/therapy suggestions that it is presenting.

### 20. I Am Responsible for My Recovery, Not the App

Although this is an item whose score would largely be a function of the user’s nature, the facilitation of user autonomy would also be conducive to this item. The app autonomy dimension 4, listed earlier in the *Eudemonic Psychology and Positive Computing* section, pertains to this item.

### 21. The More I Use the App, the More I Get Out of It

This item departs significantly from the original ARM item from which it was derived, which says that *the therapist and client are willing to work hard*. The resulting mARM item was due to a rewording following the suggestion of one participant in the research survey, given that the original version was deemed of low relevance.

The original item comes under the partnership component of the ARM, and although this modified mARM version relates to the connection between a user and an app, it does not seem to necessarily be an indicator of beneficial app use. It may very well be the case that a moderate amount of app use benefits a user, but that anything beyond this does not provide any additional therapeutic benefit; in some cases, more use could be detrimental. Therefore, even if the score for this item is not high, this does not necessarily mean that the user did not get a lot out of their connection with the app or that using the app did not result in significant, positive therapeutic change.

### 22. The App Gives Me the Confidence to Take the Lead in My Recovery

Competence, which is the second component of SDT and as discussed earlier can be fostered by positive computing factors, relates to this item. In this way, apps should include optimal challenges, positive feedback, and learning opportunities.

### 23. I Agree With the Direction the App Is Taking Me

Failures of HCI principles could contribute to lower scores for this item, for example, if an app fails to accurately tailor itself to the user or if an app operates in a way that conflicts with the user’s sense of autonomy or competence. However, responses to this item would also largely be a function of the user’s needs and the psychological content of the app.

### 24. The App Is Like Having a Member of My Care Team in My Pocket (A Novel Addition [Not Resulting From Retention or Adaption] to Capture a Key Theme From Interviews)

Two factors that will determine the score for this item are the extent to which the individual is in possession of their phone and the quality of care provided by the app. An individual being in possession of their phone for a large majority of the day and they deeming the app to be useful and supportive are likely to help the score for this item. The connection a user has with their phone, including its status as a potential attachment object, could also influence this item.

### 25. I Am Clear About What the App Can and Cannot Offer Me

The original ARM version of this item was about the therapist and client being clear about their roles and responsibilities in their interaction. Once again, this mARM version departs a fair bit from what the original item was measuring. If a user is clear about the app, this is likely to support other mARM items. For example, it is likely to reduce the chances of the user being disappointed in the app.

However, perhaps in departing from the original, this item unnecessarily removes elements that could be captured. It is certainly possible to ask whether a user is clear about their responsibilities in using and interacting with the app. On the other side, although an app cannot understand its roles and responsibilities, it can be judged on whether it was designed and functions appropriately to fulfill the roles and responsibilities it should have.

### Conclusions

The growing presence of mental health apps and digital mental health interventions in general calls for research into the notion of a DTA. The significance of the TA in traditional mental health therapy suggests the importance of considering its translation in the digital context. At this early stage, the TA does not seem to be associated with therapeutic outcomes in digital interventions as it is with human-human therapy. It may, however, be conducive to increased engagement with and adherence to digital interventions, through which it could influence outcomes. However, when making such assessments at this stage, we must be mindful of the fact that the few existing studies on measuring the DTA have either been simply copied with minimal rewording or modestly adapted existing measures of the traditional TA. Irrespective of whether such attempts demonstrate some association between their alliance measures and app outcomes, a true conceptualization of the DTA may very well require its own type of measure that fundamentally differs in certain ways from the traditional TA, a radically customized or novel measure that better fits the contours of human engagement with computers and digital interventions. Thus, although work on the DTA has been largely confined to digital mental health within the field of clinical psychology, the field of HCI can, as we have shown in this paper, profitably play a part. First, as by definition, HCI studies the interaction between humans and computers that is central to the DTA, it can be applied to determine inadequacies in simply translating traditional measures of the TA and can help to shape a novel conceptualization of the DTA. Second, tools and techniques from HCI can be employed in app development to foster items in a suitable measure of the DTA.

## Abbreviations

ARM: Agnew Relationship Measure
DTA: digital therapeutic alliance
HCI: human-computer interaction
mARM: Mobile Agnew Relationship Measure
mHealth: mobile health
MOST: moderated online social therapy
PSD: persuasive systems design
SDT: Self-Determination Theory
TA: therapeutic alliance
WAI: Working Alliance Inventory
